# The External Performance Appraisal of China Energy Regulation: An Empirical Study Using a TOPSIS Method Based on Entropy Weight and Mahalanobis Distance

**DOI:** 10.3390/ijerph15020236

**Published:** 2018-01-30

**Authors:** Zheng-Xin Wang, Dan-Dan Li, Hong-Hao Zheng

**Affiliations:** 1 School of Economics, Zhejiang University of Finance & Economics, Hangzhou 310018, China; zxwang@zufe.edu.cn (Z.-X.W.); lidandanmzli@foxmail.com (D.-D.L.); 2 Center for Research of Regulation & Policy, Zhejiang University of Finance & Economics, Hangzhou 310018, China

**Keywords:** energy regulation, sustainable development, environmental performance, multi-attribute decision making, TOPSIS, information overlap

## Abstract

In China’s industrialization process, the effective regulation of energy and environment can promote the positive externality of energy consumption while reducing negative externality, which is an important means for realizing the sustainable development of an economic society. The study puts forward an improved technique for order preference by similarity to an ideal solution based on entropy weight and Mahalanobis distance (briefly referred as E-M-TOPSIS). The performance of the approach was verified to be satisfactory. By separately using traditional and improved TOPSIS methods, the study carried out the empirical appraisals on the external performance of China’s energy regulation during 1999~2015. The results show that the correlation between the performance indexes causes the significant difference between the appraisal results of E-M-TOPSIS and traditional TOPSIS. The E-M-TOPSIS takes the correlation between indexes into account and generally softens the closeness degree compared with traditional TOPSIS. Moreover, it makes the relative closeness degree fluctuate within a small-amplitude. The results conform to the practical condition of China’s energy regulation and therefore the E-M-TOPSIS is favorably applicable for the external performance appraisal of energy regulation. Additionally, the external economic performance and social responsibility performance (including environmental and energy safety performances) based on the E-M-TOPSIS exhibit significantly different fluctuation trends. The external economic performance dramatically fluctuates with a larger fluctuation amplitude, while the social responsibility performance exhibits a relatively stable interval fluctuation. This indicates that compared to the social responsibility performance, the fluctuation of external economic performance is more sensitive to energy regulation.

## 1. Introduction

High-energy consumption, high pollution, and low energy efficiency in China have been more prominent due to various factors including extensive production modes, the absence of energy regulation, and incomplete policy execution. Although energy management practice and the improvement of energy efficiency bring about a significant marginal improvement, numerous energy-intensive enterprises do not carry out effective energy management practices due to diverse reasons such as the lack of a synergistic effect between various stakeholders and having little competitive pressure when conducting the environment-friendly management practices [1,2]. Therefore, it is necessary to perform energy regulation. Moreover, the energy industry shows a significant positive-negative externality and the energy regulation aims to promote positive externality, reducing and even eliminating the negative externality through regulations. Thus, appraising the external performance of energy regulation can provide the appraisal indexes and method for reasonably, effectively, and orderly conducting the energy regulation to further elevate the quality level of the energy regulation. This exerts a practical significance to further improvement of the utilization efficiency of industrial energies and reduction of energy intensity.

Extensive previous literatures about energy regulation mainly analyzed influences and methods of energy regulation. Scholars hardly appraise the quality level of global energy industry regulation in terms of the external performance of the energy regulation. Through empirical analysis, Cubbin and Stern pointed out that the quality level of regulation has a significant positive correlation with the productivity per capita and the utilization of productive capacity in an empirical analysis [3]. It indicates that the regulation quality of energy exerts an important effect on the implementation of the regulations. Therefore, conducting appraisal and comparative analysis on the performance level of China’s energy regulation by establishing an external performance index system of energy regulation has a practical significance to measurement and orientation of the quality level of China’s energy regulation. Additionally, as an important method for solving the multi-attribute decision making problem, the technique for order preference by similarity to an ideal solution based on the entropy weight (E-TOPSIS) is little used to appraise the performance of energy regulation. Thus, this study establishes two indexes (external economic performance and social responsibility performance) based on related data of China’s energy industry during 1995~2015. On this basis, the performance level of China’s energy regulation is appraised and compared separately using traditional TOPSIS, E-TOPSIS, and E-M-TOPSIS to analyze the development trend of the quality level of China’s energy regulation.

The rest of the study is organized as follows: Section 2 mainly introduces and reviews the literatures related to the energy regulation and TOPSIS method. Section 3 introduces traditional TOPSIS and E-M-TOPSIS, and proves the properties of the E-M-TOPSIS. Section 4 establishes an index system for external performance appraisal of the energy regulation and conducts the descriptive statistical analysis of the index data. Section 5 appraises and analyzes the index data concerning the external performance of China’s energy regulation during 1999~2015 using the appraisal methods in Section 3 to further give the corresponding policy suggestions. The Section 6 comes to a conclusion.

## 2. Literature Review

### 2.1. Research on the Energy Regulation

Energy regulation refers to a series of activities aiming to promote the positive externality while reducing and even eliminating the negative externality by implementing the regulation function in energy field. An increasing number of scholars have investigated the influence of energy regulation. For example, Matsumura et al. internalized the negative externality of energy consumptions by introducing the Pigovian tax to further analyze the influence of additional energy regulation on welfare effects [4]. Their results showed that the additional energy-conservation regulation does harm to long-term social welfares under a perfect competition market. However, under an imperfect competition market, the energy-conservation regulation reduces the cost of energy consumptions and accelerates market competition by increasing the investment of enterprises in energy conservation to future enhance the extra social welfares. Additionally, numerous scholars have explored the influence of regulations on specific energy industries. By employing an autoregressive distributed lag model (ARDL) constrained test and error correction model (ECM), Zhao et al. studied the effect of the regulation on renewable energy power generation. The research result indicated that the regulation has a significant positive effect on the development of renewable energies [5]. From the perspectives of electricity regulation and new energy, Bradshaw suggested that regulation innovation of power system reform has an important effect on overcoming the technological and institutional lock-out of wind and solar energies [6]. In terms of the regulation of energy prices, Ju et al. investigated the prices of five energies involving natural gas, gasoline, fuel oil, steam coal, and coking coal, and pointed out that the energy price distortion caused by energy price regulation is favorable for China’s economic development [7]. However, Shi and Sun shared a different point of view in their studies of China’s industrial output using the growth models of two sectors and the result showed that the regulatory price distortion exerts a negative influence on both short- and long-term output growths of China [8].

Apart from the aforementioned researches on the influence analysis of the energy regulation, specific energy regulation methods have also gradually become a research hotspot. Abrardi and Cambini suggested that an optimal tariff structure is able to drive the regulated public utilities to decrease energy consumption and enhance energy efficiency so as to obtain a low oil price for attracting consumers [9]. Under the performance-based regulation, Mandel simulated the influence of performance incentive measures on upstream energy efficiency [10]. Additionally, in order to realize energy management and energy-conservation improvement of the machine manufacturing industry, Cai et al. determined a multi-target energy benchmark by using TOPSIS to put forward a multi-target energy benchmark method based on energy-consumption prediction and comprehensive appraisal [11]. However, there are only a few literatures analyzing the regulation performance appraisal of the global energy industry. Existing literatures mainly analyze the subdivisions of the electric power industry. For example, Thamae et al. appraised the regulation performance of Lesotho’s electric power industry during 2004~2014 from the aspects of governance, substance, and impact [12].

### 2.2. Research Related to TOPSIS Appraisal Method

An external performance appraisal of China’s energy regulation is a multi-attribute decision making problem and there are numerous multi-attribute decision making methods [13,14]. Therein, the TOPSIS method is widely used in various fields such as the economy [15,16,17] and management [18,19,20] due to its characteristics including its simple principle, intuitive geometric interpretation, and the fact that it has no special requirements for sample data. As a multi-attribute decision making method, TOPSIS was first proposed by Hwang and Yoon in 1981, and improved and expanded by Zavadskas et al. and Triantaphyllou [21,22,23]. Therein, Triantaphyllou [23] pointed out that using different distance approaches for the same multi-attribute decision problem may result in different results. On this basis, Chen and Tsao [24] compared and analyzed the intuitionistic fuzzy TOPSIS results yielded by different distance approaches. Chang et al. [25] evaluated the performance of mutual funds by extended TOPSIS using two different distance approaches, namely, “Minkowski’s metric” and “Mahalanobis” distances. Furthermore, Antuchevičienė et al. [26] and Wang and Wang [27] put forward an improved TOPSIS appraisal method based on Mahalanobis distance with an aim to favorably solve the problem of a linear correlation between indexes.

Afterwards, the TOPSIS method was integrated within different weighting methods for further utilization. For example, You et al. determined the weights of indexes using the best-worst method (BWM) to establish the BMW-TOPSIS method for appraising the operation performance of power grid enterprises [20]. By combining the information entropy method to determine weights, Wang et al. and Chauhan et al. established an improved TOPSIS method to investigate the energy performance [28,29]. The use of these methods requires linear independence between various indexes when calculating the distances of various schemes to the positive and negative ideal solutions by using the Euclidean distance in the TOPSIS method. Xin et al. transformed the second-order index of the social security index system into linear independence variables using principal component analysis (PCA). On this basis, they conducted comprehensive appraisal and sorting on social security levels of 31 provinces in mainland China by the TOPSIS comprehensive appraisal method [30]. Although the PCA method can deal with the problems concerning linear correlation between indexes to some extent, it has the drawback of information loss. Thus, based on the M-TOPSIS method, the study determined the weights of various indexes by using information entropy. Afterwards, the study appraised and compared the performance levels of China’s energy regulation using E-M-TOPSIS to sufficiently analyze the development trend of the quality level of China’s energy regulation.

## 3. The Method for Appraising the External Performance of Energy Regulation

### 3.1. Traditional TOPSIS Method

TOPSIS is a widely-used method for solving uncertain multi-attribute decision making problems due to its superiorities including its rational and understandable logic, limited subjective input, and the ability to identify the best alternative quickly and incorporate relative weights of criterion importance [31,32,33,34,35]. Its ranking standard is to evaluate the distances between the appraisal objects and the positive $S^{+}$ and negative ideal $S^{-}$ solutions. Therein, the positive ideal solution is composed of optimal solutions of all indexes, while the negative ideal solution consists of the least solutions of all indexes. According to the distance between the appraisal objects and the positive $S^{+}$ and negative $S^{-}$ ideal solutions, the relative closeness is calculated, and the ranking of each scheme is then obtained. That is, the larger the $c_{i}$ is, the more optimal the scheme.

Specifically, it is assumed that there are m scheme sets $A=\{A_{1},A_{2},\ldots ,A_{m}\}$ and n index sets $F=\{f_{1},f_{2},\ldots ,f_{n}\}$ and the all indexes are divided into benefit and cost types. The decision judgment matrix $X={\left(x_{ij}\right)}_{m\times n},i=1,2,\ldots ,m;j=1,2,\ldots ,n$ is established, in which $x_{ij}$ refers to the value of the jth index in the ith scheme. The weight vectors of all indexes are $W=\{\omega_{1},\omega_{2},\ldots ,\omega_{n}\}$. The TOPSIS method used in the performance appraisal is summarized as follows [21,22,23,24,25,26]:

A. The standardized decision matrix $R={\left(r_{ij}\right)}_{m\times n}$ is built, which is then used to standardize the judgment matrix, therein,

(1) $$r_{ij}=\frac{x_{ij}}{\sqrt{\sum_{i=1}^{m}x_{ij}^{2}}}$$

B. The weighting standardized decision matrix $Z={\left(z_{ij}\right)}_{m\times n}$ is built.

(2) $$z_{ij}=\omega_{j}r_{ij}$$

C. The positive ($S^{+}$) and negative ($S^{-}$) ideal solutions are determined.

(3) $$\begin{array}{c} S^{+}=\{s_{1}^{+},s_{2}^{+},\ldots ,s_{n}^{+}\}\\ S^{-}=\{s_{1}^{-},s_{2}^{-},\ldots ,s_{n}^{-}\} \end{array}$$

For the benefit index, we obtain:

$$s_{j}^{+}=\max\{z_{ij}|1\leq i\leq m\},s_{j}^{-}=\min\{z_{ij}|1\leq i\leq m\}$$

For the cost index, we obtain:

$$s_{j}^{+}=\min\{z_{ij}|1\leq i\leq m\},s_{j}^{-}=\max\{z_{ij}|1\leq i\leq m\}$$

D. The Euclidean distances ($d_{i}^{+}$ and $d_{i}^{+}$) between each of the schemes and the positive and negative ideal solutions are separately calculated.

(4) $$d_{i}^{+}=\sqrt{\sum_{j=1}^{n}{\left(s_{j}^{+}-z_{ij}\right)}^{2}},i=1,2,\ldots ,m$$

(5) $$d_{i}^{-}=\sqrt{\sum_{j=1}^{n}{\left(s_{j}^{-}-z_{ij}\right)}^{2}},i=1,2,\ldots ,m$$

E. The relative closeness degree $c_{i}$ between each of the schemes and the positive ideal solutions is calculated:

(6) $$c_{i}=\frac{d_{i}^{-}}{d_{i}^{-}+d_{i}^{+}},i=1,2,\ldots ,m$$

F. The sorting is conducted according to the value of $c_{i}$ and obviously, the larger the $c_{i}$ is, the more optimal the scheme.

The traditional TOPSIS appraisal method can objectively reflect the difference between various appraisal schemes by introducing positive and negative ideal solutions. However, when there is a significant linear correlation between indexes, the column vector composed of n different attribute indexes cannot make up a group of bases for measuring this linear space. Therefore, some problems appear while using the Euclidean distance to calculate the distance of the various schemes to the positive and negative ideal solutions, which leads to the deviation of the final sorting results of each scheme.

### 3.2. An Improved TOPSIS Method Based on Entropy Weight and Mahalanobis Distance

Wang and Wang [27] improved the traditional TOPSIS method by introducing the Mahalanobis distance. On this basis, in order to solve the information overlap problem caused by the correlation between variables, the study further determines the weight of each index by using information entropy to establish an objective E-M-TOPSIS method for solving the multi-attribute decision making problem. Moreover, the study has verified the properties of the method.

#### 3.2.1. Definition of Mahalanobis Distance

The Mahalanobis distance is a statistical distance measure introduced by Mahalanobis, which considers the correlations of the data set and scale-invariant [27,36]. This measure is widely used in various fields such as data clustering [37,38] and multivariate diagnosis and pattern recognition [39,40].

Specifically, for a multivariate vector $x={\left(x_{1},x_{2},\ldots ,x_{n}\right)}^{T}$, mean vector $\mu ={\left(\mu_{1},\mu_{2},\ldots ,\mu_{n}\right)}^{T}$, and covariance matrix ∑, the Mahalanobis distance is:

$$D_{M}(x)=\sqrt{{\left(x-\mu \right)}^{T}\sum^{-1}(x-\mu )}$$

#### 3.2.2. E-M-TOPSIS Method 

The study improves the traditional TOPSIS method by introducing the Mahalanobis distance and further measures the weight of each index using information entropy. The Mahalanobis distance is a statistical distance characterized by independence on the measurement scale, being free from the influence of dimensions between coordinates, and capable of removing the disturbance of the correlation between variables, namely, it is able to offset the influence of linear correlation between attribute indexes. Meanwhile, information entropy can objectively and reasonably determine the weights of each of the indexes.

Suppose there is an appraisal system with m scheme sets $A=\{A_{1},A_{2},\ldots ,A_{m}\}$ and n index sets $F=\{f_{1},f_{2},\ldots ,f_{n}\}$. All indexes are divided into benefit and cost types. Following Wang and Wang [27], the improved TOPSIS method based on entropy weight and Mahalanobis distance used for the performance appraisal is illustrated in detail as follows:

A. The vector of the appraisal scheme of $A_{i}$ is constructed as follows:

$$r_{i}={\left(r_{i1},r_{i2},\ldots ,r_{in}\right)}^{T}$$

where $r_{i}$ refers to the corresponding spatial coordinate of the attribute value of the *i*th appraisal scheme. The corresponding appraisal matrix is displayed as follows:

$$R=\left[\begin{array}{cccc} r_{11} & r_{12} & \cdots & r_{1n}\\ r_{21} & r_{22} & \cdots & r_{2n}\\ \vdots & \vdots & \ddots & \vdots \\ r_{m1} & r_{m2} & \cdots & r_{mn} \end{array}\right]$$

B. Standardized processing of data

An appraisal matrix is subjected to standardized processing and therefore the following formula can be obtained:

(7) $$O={\left(o_{ij}\right)}_{m\times n},i=1,2,\ldots ,m;j=1,2,\ldots ,n,$$

where $o_{ij}$ represents the value of the jth appraisal index in the ith appraisal scheme and also $o_{ij}\in [0,1]$ and,

$$o_{ij}=\left\{ \begin{array}{l} \frac{r_{ij}-\underset{i}{\min}\{r_{ij}\}}{\underset{i}{\max}\{r_{ij}\}-\underset{i}{\min}\{r_{ij}\}},\;\mathrm{Benefit}\;\mathrm{index}\\ \frac{\underset{i}{\max}\{r_{ij}\}-r_{ij}}{\underset{i}{\max}\{r_{ij}\}-\underset{i}{\min}\{r_{ij}\}},\;\mathrm{Cos}t\;\mathrm{index} \end{array}\right.$$

C. Following Shannon and Zhang et al. [41,42], the information entropy $H_{j}$ of the appraisal index is calculated, and is shown as follows:

(8) $$H_{j}=-k\sum_{i=1}^{m}\frac{o_{ij}}{\sum_{i=1}^{m}o_{ij}}\ln\left(\frac{o_{ij}}{\sum_{i=1}^{m}o_{ij}}\right)$$

where, $k=\frac{1}{\ln m}$. On the condition that $\frac{o_{ij}}{\sum_{i=1}^{m}o_{ij}}=0$, $\frac{o_{ij}}{\sum_{i=1}^{m}o_{ij}}\ln\left(\frac{o_{ij}}{\sum_{i=1}^{m}o_{ij}}\right)=0$.

D. The entropy weight $\omega_{j}$ of the appraisal index is calculated as follows:

(9) $$\omega_{j}=\frac{1-H_{j}}{n-\sum_{j=1}^{n}H_{j}}$$

Also, $0\leq \omega_{j}\leq 1$ and $\sum_{j=1}^{n}\omega_{j}=1$.

E. The positive ($S^{+}$) and negative ($S^{-}$) ideal solutions are determined.

$S^{+}={\left\{s_{1}^{+},s_{2}^{+},\ldots ,s_{n}^{+}\right\}}^{T}$, $S^{+}={\left\{s_{1}^{-},s_{2}^{-},\ldots ,s_{n}^{-}\right\}}^{T}$ separately refer to the corresponding spatial coordinates of the positive and negative solutions, which conform to Formula (3).

For the benefit index, we obtain:

$$s_{j}^{+}=\max\{r_{ij}|1\leq i\leq m\},s_{j}^{-}=\min\{r_{ij}|1\leq i\leq m\}$$

For the cost index, we obtain:

$$s_{j}^{+}=\min\{r_{ij}|1\leq i\leq m\},s_{j}^{-}=\max\{r_{ij}|1\leq i\leq m\}$$

F. The two Mahalanobis distances ($mahal_{i}^{+}$ and $mahal_{i}^{-}$) between each scheme to positive-, and negative-, ideal solutions are calculated.

(10) $$mahal(r_{i},S^{+})=\sqrt{{\left\{r_{ij}-S_{j}^{+}\right\}}^{T}\Omega^{T}\sum^{-1}\Omega \{r_{ij}-S_{j}^{+}\}},i=1,2,\ldots ,m$$

(11) $$mahal(r_{i},S^{-})=\sqrt{{\left\{r_{ij}-S_{j}^{-}\right\}}^{T}\Omega^{T}\sum^{-1}\Omega \{r_{ij}-S_{j}^{-}\}},i=1,2,\ldots ,m$$

where $\sum^{-1}$ is the inverse matrix of the covariance matrix ∑ of attribute variables $r_{1},r_{2},\ldots ,r_{n}$, $\Omega =diag(\sqrt{\omega_{1}},\sqrt{\omega_{2}},\ldots ,\sqrt{\omega_{n}})$.

G. The relative closeness degree $c_{i}$ between each of the various schemes and the positive ideal solutions can be expressed as follows:

(12) $$c_{i}=\frac{mahal(r_{i},S^{-})}{mahal(r_{i},S^{-})+mahal(r_{i},S^{+})},i=1,2,\ldots ,m$$

H. The sorting is conducted according to the value of $c_{i}$ and the larger the $c_{i}$ is, the more optimal the scheme.

#### 3.2.3. Properties of the E-M-TOPSIS Method

The E-M-TOPSIS method has two properties.

**Property** **1.** The relative closeness degree $c_{i}$ calculated by using the E-M-TOPSIS method is unchanged for non-singular linear transformation.

**Proof of Property** **1.** It is assumed that $r_{i}={\left(r_{i1},r_{i2},\ldots ,r_{in}\right)}^{T}$ , $\tilde{r_{i}}={\left(a_{1}+b_{1}r_{i1},a_{2}+b_{2}r_{i2},\ldots ,a_{n}+b_{n}r_{in}\right)}^{T}$, $S^{+}={\left\{s_{1}^{+},s_{2}^{+},\ldots ,s_{n}^{+}\right\}}^{T}$, and ${\tilde{S}}^{+}={\left(a_{1}+b_{1}s_{1}^{+},a_{2}+b_{2}s_{2}^{+},\ldots ,a_{n}+b_{n}s_{n}^{+}\right)}^{T}$. Here, $a_{i}$ and $b_{i}$ are constants and $b_{i}\neq 0$. Assuming that $A={\left(a_{1},a_{2},\ldots ,a_{n}\right)}^{T}$, $B=diag(b_{1},b_{2},\ldots ,b_{n})$, and then $\tilde{r_{i}}=A+Br_{i}$, ${\tilde{S}}^{+}=A+BS^{+}$. For the condition $\tilde{\sum }=B\sum B^{T}$, ${\tilde{\sum }}^{-1}={\left(B^{-1}\right)}^{T}\sum^{-1}B^{-1}$, and therefore:

$$\begin{array}{ll} mahal(\tilde{r_{i}},{\tilde{S}}^{+}) & =\sqrt{{\left(\tilde{r_{i}}-{\tilde{S}}^{+}\right)}^{T}\Omega^{T}{\tilde{\sum }}^{-1}\Omega (\tilde{r_{i}}-{\tilde{S}}^{+})}\\ & =\sqrt{{\left(A+Br_{i}-A-BS^{+}\right)}^{T}\Omega^{T}{\left(B^{-1}\right)}^{T}\sum^{-1}B^{-1}\Omega (A+Br_{i}-A-BS^{+})}\\ & =\sqrt{{\left(r_{i}-S^{+}\right)}^{T}B^{T}\Omega^{T}{\left(B^{-1}\right)}^{T}\sum^{-1}B^{-1}\Omega B(r_{i}-S^{+})}\\ & =\sqrt{{\left(r_{i}-S^{+}\right)}^{T}\Omega^{T}\sum^{-1}\Omega (r_{i}-S^{+})}=mahal(r_{i},S^{+}) \end{array}$$

Similarly, $mahal(\tilde{r_{i}},{\tilde{S}}^{-})=mahal(r_{i},S^{-})$.

Therefore, the relative closeness degree through the non-singular transformation can be expressed as follows:

$$\tilde{c_{i}}=\frac{mahal(\tilde{r_{i}},{\tilde{S}}^{-})}{mahal(\tilde{r_{i}},{\tilde{S}}^{-})+mahal(\tilde{r_{i}},{\tilde{S}}^{+})}=\frac{mahal(r_{i},S^{-})}{mahal(r_{i},S^{-})+mahal(r_{i},S^{+})}$$

Property 1 indicates that if the standardization of the original data is a non-singular transformation during the decision making, the standardized process cannot affect the decision-making result.

**Property** **2.** On the condition that the appraisal indexes $f_{1},f_{2},\ldots ,f_{n}$ show linear independence.

$$mahal(r_{i},S^{+})=\sqrt{\sum_{j=1}^{n}\frac{\omega_{j}{\left(r_{ij}-s_{j}^{+}\right)}^{2}}{\sigma_{j}^{2}}}\text{ and }mahal(r_{i},S^{-})=\sqrt{\sum_{j=1}^{n}\frac{\omega_{j}{\left(r_{ij}-s_{j}^{-}\right)}^{2}}{\sigma_{j}^{2}}}$$

**Proof of Property** **2.** It is assumed that $r_{i}$ and $S^{+}$ are taken from the same n-dimensional appraisal system, where the mean is $\mu ={\left(\mu_{1},\mu_{2},\ldots ,\mu_{n}\right)}^{T}$ and the covariance is ∑. The weight vector of the indexes is W and $\Omega =diag(\sqrt{\omega_{1}},\sqrt{\omega_{2}},\ldots ,\sqrt{\omega_{n}})$. Due to the linear independence between various indexes, $\sum =diag(\sigma_{1}^{2},\sigma_{2}^{2},\ldots ,\sigma_{n}^{2})$ and $\sum^{-1}=diag\left(\frac{1}{\sigma_{1}^{2}},\frac{1}{\sigma_{2}^{2}},\ldots ,\frac{1}{\sigma_{n}^{2}}\right)$,

$$\begin{array}{ll} mahal^{2}(r_{i},S^{+}) & ={\left\{r_{ij}-S_{j}^{+}\right\}}^{T}\Omega^{T}\sum^{-1}\Omega \{r_{ij}-S_{j}^{+}\}\\ & =(\sqrt{\omega_{1}}(r_{i1}-s_{1}^{+}),\ldots ,\sqrt{\omega_{n}}(r_{in}-s_{n}^{+}))\times \left[\begin{array}{ccc} \frac{1}{\sigma_{1}^{2}} & & \\ & \ddots & \\ & & \frac{1}{\sigma_{n}^{2}} \end{array}\right]\left(\begin{array}{c} \sqrt{\omega_{1}}(r_{i1}-s_{1}^{+})\\ \vdots \\ \sqrt{\omega_{n}}(r_{in}-s_{n}^{+}) \end{array}\right)\\ & =\sum_{j=1}^{n}\frac{\omega_{j}{\left(r_{ij}-s_{j}^{+}\right)}^{2}}{\sigma_{j}^{2}} \end{array}$$

Therefore, $mahal(r_{i},S^{+})=\sqrt{\sum_{j=1}^{n}\frac{\omega_{j}{\left(r_{ij}-s_{j}^{+}\right)}^{2}}{\sigma_{j}^{2}}}$.

Similarly, $mahal(r_{i},S^{-})=\sqrt{\sum_{j=1}^{n}\frac{\omega_{j}{\left(r_{ij}-s_{j}^{-}\right)}^{2}}{\sigma_{j}^{2}}}$.□

Thus, when the appraisal indexes are independent of each other, the weighted Mahalanobis distance is equivalent to the weighted Euclidean distance. However, when the appraisal indexes are correlated with each other, the Mahalanobis distance is shown to be little influenced by the dimension of indexes. Meanwhile, it is able to eliminate information overlap caused by the linear correlation between indexes. Therefore, the Mahalanobis distance is more applicable for solving the complex practical problems. Additionally, in practical applications, the general covariance matrix is unknown and therefore it can be replaced by a sample covariance matrix.

In conclusion, the properties, advantages, and limitations of traditional TOPSIS, E-TOPSIS, and E-M-TOPSIS are shown in Table 1.

## 4. Appraisal Indexes and Data Concerning External Performances of Energy Regulation

### 4.1. The Appraisal Indexes Concerning External Performance of Energy Regulation

The study selected and constructed performance indexes concerning the external responsibility of energy regulation based on a result-oriented principle [43]. The result-oriented principle is one of the basic concepts and core ideas of the performance management theory, which emphasizes the results of operation, management, and work, namely, economic and social benefits, as well as customer satisfaction. The result-oriented principle for the external performance appraisal of energy regulation also intensively analyzes the economic and social benefits, as well as the public’s satisfaction degree caused by energy regulation. Considering the loss of corresponding data of the public’s satisfaction degree of energy regulation, the study divided performance indexes concerning external responsibility of energy regulation into external economic performance and social responsibility performance for selection and establishment.

The economic performance refers to the efficiency appraisal of the resource allocation and utilization. Following Wang [44], the external economic performance of energy regulation mainly involves four indexes: energy consumption elasticity coefficient, power consumption elasticity coefficient, outputs of energy, and power consumptions per unit. In detail, the energy and power consumption elasticity coefficients separately refer to ratios of the growth rates of energy and power consumptions to that of the national economy. This reflects the structural relationship between the development rate of the national economy and the energy or power consumption. The outputs of the energy and power consumptions per unit separately denote the Gross Domestic Product (GDP) produced by the energy or power consumption per unit of a country or a region within a certain period. The two indexes reflect the utilization degree and output efficiency of the energy or the power in economic activities of a country or a region.

The performance index about social responsibility mainly involves indexes concerning environmental performance and energy safety related to the energy consumption. The environmental performance represents the negative external effect on society during the energy utilization whose specific indexes include SO_2_ emission amount per GDP, dust emission amount per GDP, and wastewater discharge amount per GDP. These indexes reflect the influence of energy utilization on the environment. The energy safety performance mainly deals with the core problem of energy safety: whether the energy supply is sufficient and stable or not, and its specific indexes include external dependence, the proportion of primary energy yield in the total world yield, and the primary energy self-sufficient rate. Here, the external dependence reflects the correlation degree of a country on the foreign energies, while the proportion of the primary energy yield in the worldwide yield and the primary energy self-sufficient rate both show the supply capability of China’s energies.

Overall, this study establishes the performance index system for the external responsibility of energy regulation, as shown in Table 2. Here, the output of energy consumption per unit $\left(X_{3}\right)$, output of power consumption per unit $\left(X_{4}\right)$, proportion of primary energy yield in the worldwide yield $\left(X_{9}\right)$, and primary energy self-sufficient rate $\left(X_{10}\right)$ are separate benefit indexes. The energy consumption elasticity index $\left(X_{1}\right)$, power consumption elasticity index $\left(X_{2}\right)$, SO_2_ emission amount per GDP $\left(X_{5}\right)$, dust emission amount per GDP $\left(X_{6}\right)$, wastewater discharge amount per GDP $\left(X_{7}\right)$, and external dependence $\left(X_{8}\right)$ are all cost indexes.

### 4.2. Descriptive Statistical Analysis 

The data are taken from China Stock Market & Accounting Research (CSMAR) database, Wind database, and annual *China Energy Statistical Yearbook* (In detail, the data about energy consumption elasticity coefficients, power consumption elasticity coefficients, total energy consumptions, import volumes of energies, the total power consumptions, GDPs, GDP deflators, SO_2_ and dust emission amounts, and wastewater discharge amount from 1999~2015 are taken from the CSMAR database. The data about the yields, import volumes, and consumptions of the primary energy during 1999~2015 are collected from the Wind database. The total world energy yields during 1999~2015 are taken from the yearly *China Energy Statistical Yearbook*. It is worth noting that the total world energy yield in 2015 was not recorded because the *China Energy Statistical Yearbook* of 2017 has not been published. The study acquired the total world energy yield in 2015 by measuring the average growth rate of the total world energy yields in the most recent five years from 2010 to 2014. Additionally, the GDP is calculated according to the GDP deflators by taking 1999 as the base period.). All index data have been subjected to a descriptive statistical analysis and the specific descriptive statistical results are shown in Table 3.

**Figure 1:**
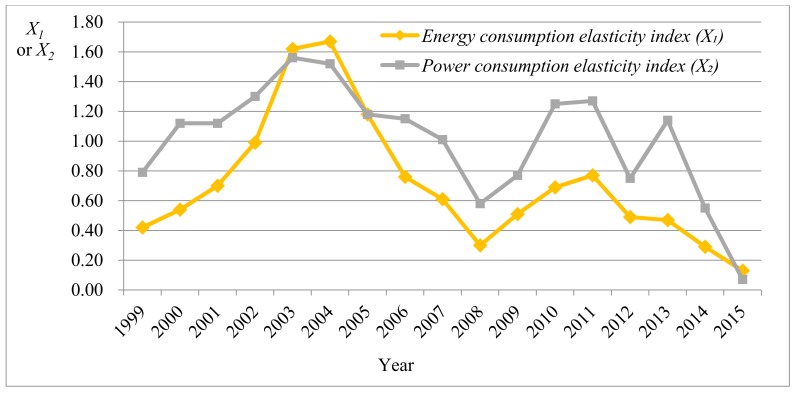
Fluctuation trend of performance indexes concerning external economic (*X*_1_ and *X*_2_).

**Figure 2:**
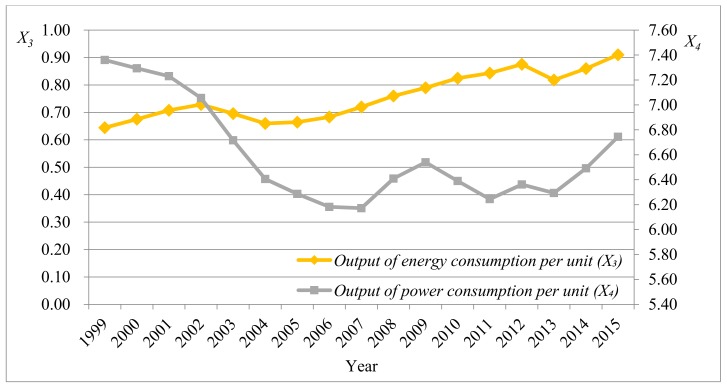
Fluctuation trend of performance indexes concerning external economic (*X*_3_ and *X*_4_).

**Figure 3:**
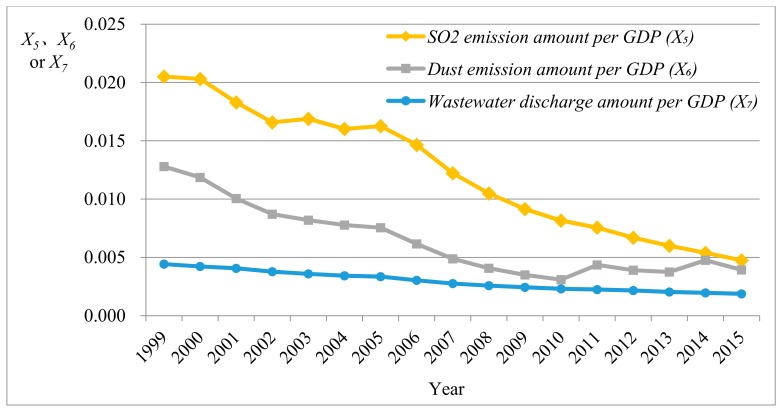
Fluctuation trend of social responsibility performance indexes (*X*_5_–*X*_7_).

**Figure 4:**
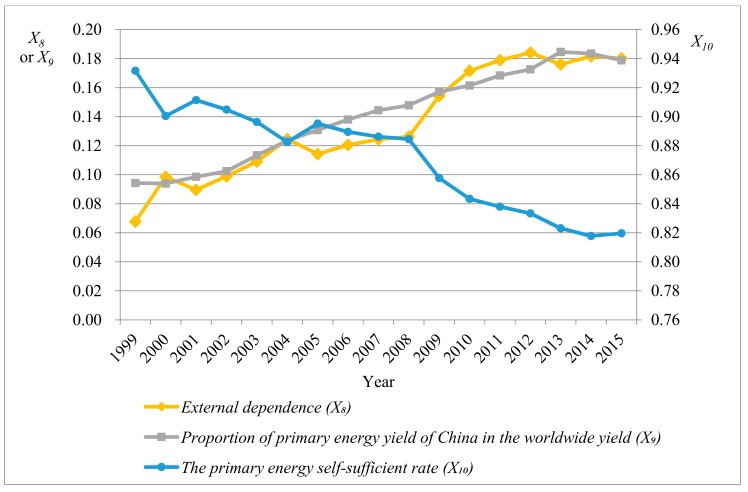
Fluctuation trend of social responsibility performance indexes (*X*_8_–*X*_10_).

It can be seen that the maximum and minimum of all indexes existed within a reasonable interval and the mean of indexes was far larger than the standard deviation, implying that there was a low dispersion degree of data. Moreover, the probability with the extreme outlier was at a low level. The mean, median, and mode of the dust emission amount per GDP $\left(X_{6}\right)$ were close to those of the wastewater discharge amount per GDP $\left(X_{7}\right)$, indicating that the data of the two indexes were approximately symmetrically distributed. It can be speculated from the skewness that the data of power consumption elasticity index $\left(X_{2}\right)$, external dependence $\left(X_{8}\right)$, proportion of primary energy yield in the worldwide yield $\left(X_{9}\right)$, and primary energy self-sufficient rate $\left(X_{10}\right)$ were left-skewed distributed. The other index data were right-skewed distributed. Figure 1, Figure 2, Figure 3 and Figure 4 separately display fluctuation trends of performance indexes concerning external economic and social responsibility.

As shown in Figure 1, the fluctuation trend of the energy consumption elasticity index $\left(X_{1}\right)$ was similar to that of the power consumption elasticity index $\left(X_{2}\right)$ overall. The two indexes both rose to a peak around 2003, then gradually declined, before increasing after reaching the minimum in 2008 (global financial crisis), and finally reached the peak in 2011. Following this, a new cycle began.

In Figure 2, the output of energy consumption per unit $\left(X_{3}\right)$ generally exhibiting a rising trend except for the slight decrease in 2003 and 2013, which indicated that China’s energy utilization rate was increasingly high and the output efficiency significantly improved. The output of power consumption per unit $\left(X_{4}\right)$ constantly decreased from 1999 to the minimum in 2007, then fluctuated and constantly rose from 2013.

It can be seen from Figure 3 that SO_2_ emission amount per GDP $\left(X_{5}\right)$, dust emission amount per GDP $\left(X_{6}\right)$, and wastewater discharge amount per GDP $\left(X_{7}\right)$ (environmental performance index) decreased year by year. This implied that China pays more attention to environmental protection while consuming plenty of energy. Moreover, in Figure 4, although the proportion of primary energy yield of China in the worldwide yield $\left(X_{9}\right)$ basically improved year by year, the primary energy self-sufficient rate $\left(X_{10}\right)$ declined overall and the external dependence $\left(X_{8}\right)$ significantly rose. This indicated that China’s energy consumption is still greatly increasing while the domestic energy supply capacity cannot satisfy the rapidly growing energy demand.

Moreover, all index data were subjected to the Pearson correlation analysis for testing the correlation between indexes, and the correlation results are shown in Table 4.

It can be seen that a common correlation between various performance indexes exists and 71% of correlation coefficients between indexes show significant statistics under 5% of the significant level. Especially, the indexes including output of energy consumption per unit $\left(X_{3}\right)$ and SO_2_ emission amount per GDP $\left(X_{5}\right)$ basically show a significant correlation relationship with all the other indexes. Therefore, during selecting the methods for performance appraisal, it is necessary to select a proper method for solving the correlation in order to avoid the information overlap problem.

## 5. Empirical Results of the External Performance Appraisal of China Energy Regulation

### 5.1. The External Performance Appraisal of China Energy Regulation Based on the E-M-TOPSIS Method

Based on the designed appraisal method for the external performance of energy regulations and selected appraisal indexes, the study evaluates the external performance of China’s energy regulation using the E-M-TOPSIS.

Firstly, this study calculated information entropies of various indexes according to Formulas (7) and (8) to further determine the weights of the appraisal indexes by analyzing the information entropies in Formula (9). The various weights are represented as weight vector W. Therein,

W={0.055 0.092 0.116 0.158 0.098 0.057 0.076 0.135 0.110 0.104}

Afterwards, the decision judgment matrix is established to determine the positive ($S^{+}$) and negative ($S^{-}$) ideal solutions of different indexes. Here:

$$S^{+}=\{0.130\;0.070\;0.910\;7.630\;0.005\;0.003\;0.002\;0.068\;0.185\;0.932\}$$

$$S^{-}=\{1.670\;1.560\;0.644\;6.172\;0.021\;0.013\;0.004\;0.184\;0.094\;0.818\}$$

Finally, the study separately calculated the Mahalanobis distances between $r_{i}$ and $S^{+}$, as well as $r_{i}$ and $S^{-}$, and then the relative closeness degrees according to Formula (12). The results are displayed in Table 5. Additionally, there are differences between the appraisal results obtained using different appraisal methods for external performances of energy regulations. In order to compare the differences, the study also displays appraisal results of the external performances of China’s energy regulation obtained using the E-TOPSIS method in Table 5.

**Figure 5:**
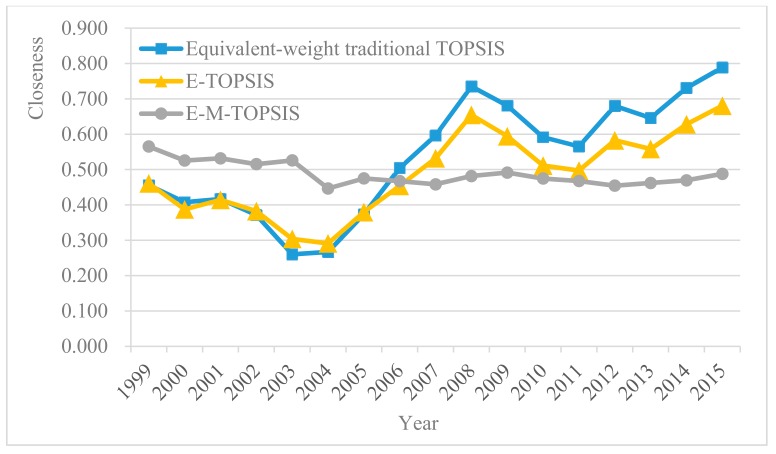
Fluctuation trends of relative closeness degrees of the equivalent-weight traditional TOPSIS, E-TOPSIS, and E-M-TOPSIS methods.

It can be speculated from the table that the presence of the correlation between indexes leads to sorting results of the external performance of energy regulations calculated using two different appraisal methods that havei a significant disparity. The E-M-TOPSIS method considering the correlation between the performance indexes concerning external economic and social responsibility exhibits a lower relative closeness degree compared with the E-TOPSIS method due to avoiding the information overlap problem. This also implies that the correlation between indexes cannot be ignorable to some extent. Therefore, due to taking the correlation between various performance indexes into account, the M-TOPSIS method can truly show the external performance characteristic of the energy regulation and reflect the performance level of energy regulation. On this basis, the method can be used for scientific decision-making formulations. Figure 5 shows the fluctuation trends of corresponding relative closeness degrees of three different appraisal methods for the external performance of energy regulation.

**Figure 6:**
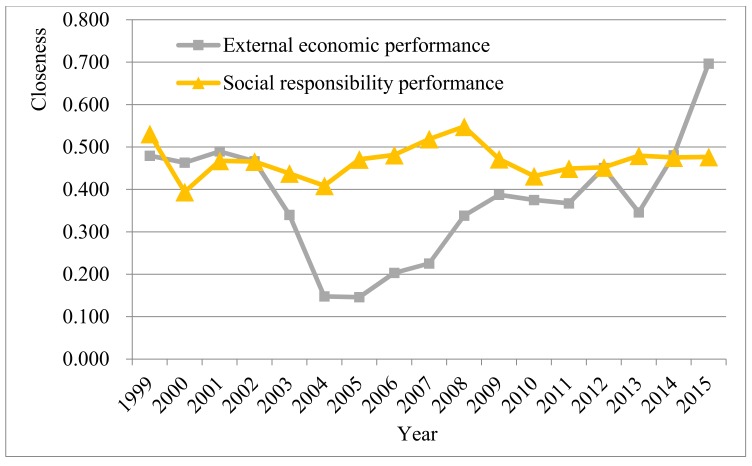
The fluctuation trends of the external economic and social responsibility performances using E-M-TOPSIS.

As shown in Figure 5, the traditional TOPSIS method enlarges the fluctuation interval of the relative closeness degree and increases the fluctuation amplitude of relative closeness degree to some extent because it cannot effectively address the information overlap problem. However, the E-M-TOPSIS method avoids information overlap and causes the relative closeness degree within a fluctuation interval to have a lower amplitude. Meanwhile, the method softens the closeness level to further acquire performance appraisal results reflecting the true level of energy regulation based on independent performance indexes. Figure 6 shows fluctuation trends of external economic and social responsibility performances obtained using the E-M-TOPSIS method. To be specific, the external economic performance dramatically fluctuates with a great fluctuation amplitude and it rose rapidly and unevenly after reaching the wave trough (the minimum value) around 2004 and 2005 (year). Moreover, the increase sped up in 2013 with the constantly deepening reform of China’s energy regulation institutions. However, the social responsibility performance maintained a relatively stable fluctuation with an interval. It can be seen that the fluctuation of the external economic performance is more sensitive to energy regulation than the social responsibility performance.

Additionally, by comparing the appraisal results of the E-TOPSIS method and the equivalent-weighting traditional TOPSIS method, it can be seen that determining weights by using information entropy does reflect its reasonable and objective characteristics to some extent. Therefore, the E-M-TOPSIS method is applicable for the external performance appraisal of the energy regulation after solving the correlation problem between indexes and determining weights through information entropy. This exerts a practical significance on the scientific appraisal and decision-making of energy regulation policies.

### 5.2. Discussion and Policy Implications 

Compared with the traditional TOPSIS method, the E-M-TOPSIS method is more applicable for evaluating the practical condition of the external performance level of China’s energy regulation, which can provide profound policy enlightenment for the management practice of energy regulation.

As shown in the fluctuation trend of the relative closeness degree using the E-M-TOPSIS method in Figure 5, the external performance of China’s energy regulation stably fluctuated within an interval overall and China’s quality level of energy regulation remained stable during 1999~2005. To be specific, the external performance of China’s energy regulation unevenly rose after reaching the low ebb in 2004, which conformed to the following fact: corresponding institutional and regulatory organizations of China’s Electricity Regulatory Commission were successively built and gradually became mature, and Regional Electricity Regulatory Bureaus were then successively established in 2004. Additionally, China’s energy institutional reform deepened in 2013 and China’s Electricity Regulatory Commission was officially merged into the National Energy Administration of the People’s Republic of China. This resulted in mode transformation from separation to union between governments and regulation. Moreover, energy regulatory content was transformed from the electricity regulation to a broad range of energy regulations (the regulations cover the areas including electricity, coal, oil, and new energy). As shown in Figure 5, the external performance level of China’s energy regulation constantly rose from 2013, which implied that a big energy regulation system of union between governments and regulation is applicable for the development phase of China’s energy field. Thus, the quality level of China’s energy regulation can be favorably elevated by promoting the energy institutional reform, perfecting the legal system and executive system under the broad range of energy regulations, and guaranteeing the steady operation of the energy regulation system.

Additionally, the weight of the performance index system for external responsibility of energy regulation based on the information entropy can be determined. The output of energy consumption per unit is shown to be the most important index influencing the external performance level of energy regulations, which reflects the utilization degree and output efficiency of energies in economic activities. Therefore, it is also an orientation index affecting the utilization efficiency and intensity of energies. Hence, improving the utilization efficiency of energies and reducing the energy intensity both exert a direct effect on improving and enhancing the external performance level of energy regulation and vice versa.

## 6. Conclusions 

The energy industry exhibits significant positive and negative externalities and the purpose of energy regulation is to promote the positive externality, reducing and even eliminating negative externality by implementing regulations. Appraising the external performance of China energy regulation will provide appraisal indexes and methods for reasonably, effectively, and orderly conducting the energy regulation and has a practical significance to further improving the quality level of energy regulations. The external performance appraisal of China’s energy regulation involves multi-attribute decision making in essence. However, inconsistent with the practical data, existing multi-attribute appraisal methods assume that the sample data are all independent and identically distributed. Therefore, in order to avoid the information overlap resulting from the correlation of indexes, the study evaluated the external performance of China’s energy regulation using the E-M-TOPSIS method. The appraisal results indicate that the presence of the correlation between indexes causes a great difference of appraised external performance levels of China’s energy regulation between the E-M-TOPSIS and traditional TOPSIS method. Compared with the traditional TOPSIS method, the E-M-TOPSIS method that considers the correlation between indexes softens the closeness level overall and causes the closeness to fluctuate within a small-amplitude interval. The appraisal result obtained using the E-M-TOPSIS method is consistent with the practical condition of China’s energy regulation. Moreover, the E-M-TOPSIS method is favorably applicable in the external performance appraisal of energy regulation, which exerts a practical significance to the scientific appraisal and decision making of energy regulation policies.

### Future Work

The study appraises and analyzes the performance level of China’s energy regulation from the aspect of external performances. Another important factor influencing the performance level of energy regulation is the fact that the indexes related to the internal performance are not introduced into the performance index appraisal system of this research due to the limits of availability and completeness of data, which will be primarily considered in the appraisal of the performance level of China’s energy regulation in the future.

## Figures and Tables

**Table 1:** The properties, advantages, and limitations of traditional TOPSIS, E-TOPSIS, and E-M-TOPSIS.

Methods	Properties	Advantages	Limitations
Traditional TOPSIS	The relative closeness degree is changed for non-singular linear transformation	1.Rational and understandable logic 2. Limited subjective input 3.The ability to identify the best alternative quickly and incorporate relative weights of criterion importance	1. Subjective weight-determining process 2. The correlation between indexes cannot be eliminated
E-TOPSIS	The relative closeness degree is unchanged for non-singular linear transformation	1. Objective weight-determining process 2. Other advantages are the same as traditional TOPSIS	The correlation between indexes cannot be eliminated
E-M-TOPSIS	1. The relative closeness degree is unchanged for non-singular linear transformation 2.When the appraisal indexes are independent of each other, the weighted Mahalanobis distance is equivalent to the weighted Euclidean distance	1. Objective weight-determining process 2. Scale-invariant property 3. Elimination of the linear correlation among indicators 4. Other advantages are the same as traditional TOPSIS	The nonlinear correlation between indexes cannot be eliminated

**Table 2:** The performance index system for external responsibility of the energy regulation.

Class		Index	Calculation Method	Unit
External economic performance		Energy consumption elasticity index $\left(X_{1}\right)$	Average annual growth rate of energy consumptions/average annual growth rate of GDP	No
		Power consumption elasticity index $\left(X_{2}\right)$	Average annual growth rate of power consumptions/average annual growth rate of GDP	No
		Output of energy consumption per unit $\left(X_{3}\right)$	GDP/total energy consumption	10^4^ CNY/tons standard coal
		Output of power consumption per unit $\left(X_{4}\right)$	GDP/total power consumptions	CNY/kW·h
Social responsibility performance	Environmental performance	SO_2_ emission amount per GDP $\left(X_{5}\right)$	SO_2_ emission amount/GDP	Tons/10^4^ CNY
		Dust emission amount per GDP $\left(X_{6}\right)$	Dust emission amount/GDP	Tons/10^4^ CNY
		Wastewater discharge amount per GDP $\left(X_{7}\right)$	Wastewater discharge amount/GDP	Tons/CNY
	Energy safety performance	External dependence $\left(X_{8}\right)$	Energy import amount/total energy consumption	No
		Proportion of primary energy yield in the worldwide yield $\left(X_{9}\right)$	Primary energy yield/total world energy yield	No
		Primary energy self-sufficient rate $\left(X_{10}\right)$	$1-\frac{\mathrm{Primary energy import}}{\mathrm{Primary energy consumption}}$	No

**Table 3:** Descriptive statistics.

Index	Mean	Median	Mode	Standard Deviation	Minimum	Maximum	Skewness	Kurtosis
Energy consumption elasticity index $\left(X_{1}\right)$	0.714	0.610	0.130 ^a^	0.433	0.130	1.670	1.154	0.875
Power consumption elasticity index $\left(X_{2}\right)$	1.008	1.120	1.120	0.380	0.070	1.560	−0.857	0.865
Output of energy consumption per unit $\left(X_{3}\right)$	0.756	0.729	0.644 ^a^	0.085	0.644	0.910	0.368	−1.276
Output of power consumption per unit $\left(X_{4}\right)$	6.599	6.409	6.172 ^a^	0.400	6.172	7.360	0.925	−0.547
SO_2_ emission amount per GDP $\left(X_{5}\right)$	0.012	0.012	0.005	0.005	0.005	0.021	0.051	−1.535
Dust emission amount per GDP $\left(X_{6}\right)$	0.006	0.005	0.003	0.003	0.003	0.013	0.851	−0.394
Wastewater discharge amount per GDP $\left(X_{7}\right)$	0.003	0.003	0.002	0.001	0.002	0.004	0.373	−1.277
External dependence $\left(X_{8}\right)$	0.135	0.125	0.068 ^a^	0.038	0.068	0.184	−0.035	−1.314
Proportion of primary energy yield in the worldwide yield $\left(X_{9}\right)$	0.141	0.144	0.094 ^a^	0.032	0.094	0.185	−0.169	−1.387
Primary energy self-sufficient rate $\left(X_{10}\right)$	0.872	0.885	0.818 ^a^	0.036	0.818	0.932	−0.218	−1.276

Note: the superscript ^a^ refers to where the index shows several modes to present the minimum of the modes in this context.

**Table 4:** Pearson correlation.

	$X_{1}$	$X_{2}$	$X_{3}$	$X_{4}$	$X_{5}$	$X_{6}$	$X_{7}$	$X_{8}$	$X_{9}$	$X_{10}$
$X_{1}$	1	0 .835 **	− 0 .559 *	− 0 .094	0 .487 *	0 .295	0 .423	− 0 .350	− 0 .440	0 .397
$X_{2}$	0 .835 **	1	− 0 .563 *	− 0 .060	0 .504 *	0 .316	0 .455	− 0 .356	− 0 .458	0 .408
$X_{3}$	− 0 .559 *	− 0 .563 *	1	− 0 .334	− 0 .932 **	− 0 .768 **	− 0 .873 **	0 .908 **	0 .859 **	− 0 .916 **
$X_{4}$	− 0 .094	− 0 .060	− 0 .334	1	0 .609 **	0 .798 **	0 .718 **	− 0 .625 **	− 0 .708 **	0 .539 *
$X_{5}$	0 .487 *	0 .504 *	− 0 .932 **	0 .609 **	1	0 .917 **	0 .986 **	− 0 .961 **	− 0 .979 **	0 .954 **
$X_{6}$	0 .295	0 .316	− 0 .768 **	0 .798 **	0 .917 **	1	0 .955 **	− 0 .873 **	− 0 .914 **	0 .819 **
$X_{7}$	0 .423	0 .455	− 0 .873 **	0 .718 **	0 .986 **	0 .955 **	1	− 0 .954 **	− 0 .988 **	0 .933 **
$X_{8}$	− 0 .350	− 0 .356	0 .908 **	− 0 .625 **	− 0 .961 **	− 0 .873 **	− 0 .954 **	1	0 .956 **	− 0 .986 **
$X_{9}$	− 0 .440	− 0 .458	0 .859 **	− 0 .708 **	− 0 .979 **	− 0 .914 **	− 0 .988 **	0 .956 **	1	− 0 .946 **
$X_{10}$	0 .397	0 .408	− 0 .916 **	0 .539 *	0 .954 **	0 .819 **	0 .933 **	− 0 .986 **	− 0 .946 **	1

Note: ** exhibits a significant correlation under a level of 0.01 (bilateral). * shows a significant correlation under a level of 0.05 (bilateral).

**Table 5:** A comparison between the appraisal results separately obtained based on E-M-TOPSIS and E-TOPSIS methods.

Year	Mahal+	Mahal−	E-M-TOPSIS		E-TOPSIS		Traditional TOPSIS	
			Closeness	Order	Closeness	Order	Closeness	Order
1999	10.307	13.397	0.565	1	0.461	10	0.456	11
2000	11.416	12.644	0.526	4	0.387	13	0.408	13
2001	11.044	12.544	0.532	2	0.414	12	0.417	12
2002	11.482	12.202	0.515	5	0.383	14	0.371	15
2003	11.198	12.420	0.526	3	0.304	16	0.260	17
2004	13.080	10.555	0.447	17	0.291	17	0.267	16
2005	12.302	11.128	0.475	9	0.380	15	0.374	14
2006	12.530	10.987	0.467	13	0.454	11	0.504	10
2007	12.753	10.783	0.458	15	0.532	7	0.596	7
2008	12.198	11.329	0.482	8	0.655	2	0.735	2
2009	12.016	11.607	0.491	6	0.594	4	0.680	4
2010	12.398	11.204	0.475	10	0.511	8	0.592	8
2011	12.587	11.057	0.468	12	0.497	9	0.565	9
2012	12.860	10.713	0.454	16	0.583	5	0.680	5
2013	12.631	10.854	0.462	14	0.558	6	0.646	6
2014	12.442	11.015	0.470	11	0.628	3	0.731	3
2015	11.999	11.433	0.488	7	0.681	1	0.788	1
